# Transactions of the Medical Association of the State of Alabama

**Published:** 1903-10

**Authors:** 


TRANSACTIONS OF THE MEDICAL ASSOCIATION OF THE STATE OF ALABAMA. Published by the Brown Printing Co., Montgomery, Ala.

We have reason to congratulate the profession of our sister State on its excellent work for the past year, as shown by the reports read at the annual State meeting.

The volume contains beside the routine reports of the committees, the excellent annual oration delivered by Dr. C. L. Morris, and various papers of merit by the most prominent doctors of the State. There is much material in it for profitable reading.

